# Selective Susceptibility of Oligodendrocytes to Carbon Monoxide Poisoning: Implication for Delayed Neurologic Sequelae (DNS)

**DOI:** 10.3389/fpsyt.2020.00815

**Published:** 2020-08-13

**Authors:** Xiaofei Tian, Teng Guan, Ying Guo, Guohui Zhang, Jiming Kong

**Affiliations:** ^1^ Department of Forensic Medicine, Hebei North University, Zhangjiakou, China; ^2^ Department of Human Anatomy and Cell Science, University of Manitoba, Winnipeg, MB, Canada

**Keywords:** carbon monoxide, cytotoxicity, oligodendrocyte, demyelination, delayed neurologic sequelae

## Abstract

Delayed neurologic sequelae (DNS) are recurrent–transient neuropsychiatric consequences of carbon monoxide (CO) intoxication. Pathologically DNS features damages to the brain white matter. Here we test a hypothesis that direct cytotoxicity of CO to oligodendrocytes plays a role in the development of DNS. In an *in vitro* model of CO poisoning with the carbon monoxide releasing molecule-2 (CORM-2) as a CO donor, we show that CORM-2 at concentrations higher than 200 µM significantly inhibited viability and caused significant death of PC12 cells. Similar minimum toxicity concentration was observed on primary brain cells including neurons, astrocytes, and microglia. Interestingly, oligodendrocytes showed cytotoxicity to CORM-2 at a much lower concentration (100 µM). We further found that CORM-2 at 100 µM inhibited proteolipid protein (PLP) production and reduced myelin coverage on axons in an *in vitro* model of myelination. Our results show that direct cytotoxicity is a mechanism of CO poisoning and DNS may result from a high susceptibility of oligodendrocytes to CO poisoning.

## Introduction

Carbon monoxide (CO) poisoning is one of the leading causes of death and injury worldwide (1). Acute symptoms of CO poisoning are non-specific and varied but are mainly associated with the brain and heart, including headache, fatigue, malaise, “trouble thinking”, confusion, nausea, dizziness, visual disturbances, chest pain, shortness of breath, loss of consciousness, and seizures. Because the brain and the heart have a high demand for oxygen, these acute symptoms are believed to be resulted from CO-induced hypoxia due to the formation of carboxyhemoglobin (COHb), which is over 200-fold more stable than oxyhemoglobin (O_2_Hb) (2, 3). Severe CO poisoning can be fatal, although mortality among patients admitted to hospitals is relatively low (4). Up to a third of survivors develop delayed neurological sequelae (DNS) within weeks after an initial complete clinical recovery from acute poisoning. Most frequently described sequelae include a broad spectrum of neurological deficits, cognitive impairments, and affective disorders. DNS gradually resolves over the first months but can be permanent in about 25% of cases. Neuroimaging studies reveal cerebral white matter hyperintensities, with delayed leukoencephalopathy in patients with DNS (5).

The mechanism by which DNS develops following CO poisoning is not fully understood. Since hypoxia alone fails to explain many pathological aspects of the DNS, it is likely that CO has direct toxicity to cells. Here we show that in normoxia CO at high concentrations is toxic to cells. Oligodendrocytes, the myelin-forming cells in the brain, are particularly susceptible to CO poisoning. Our results provide evidence to support that direct toxicity of CO to oligodendrocytes plays a role in the development of DNS.

## Materials and Methods

### Reagents and Supplies

The following reagents and supplies were commercially obtained: Dulbecco’s modified Eagle’s media/F12 (DMEM/F12; Hyclone SH30023), Dulbecco’s modified Eagle’s media (DMEM; Invitrogen/Gibco 11960), fetal bovine serum (FBS; Hyclone SV30087), insulin (Sigma, I6634), N-acetyl-l-cysteine (NAC; AMERSCO, 0LA0011), penicillin/streptomycin (Invitrogen, 15140), PDGF-AA and bFGF (PeproTech, Rocky Hill, NJ, USA), DNase I (Sigma, D5025), poly-d-lysine (PDL; Sigma, P7405), thiazolyl blue tetrazolium bromide (MTT; Sigma, M5655), Hoechst 33258 (Sigma, 94403), propidium iodide (PI; Sigma, P4170), Dulbecco’s phosphate-buffered saline (DPBS) without Mg and Ca (Invitrogen 14190-144), and Dimethyl Sulfoxide (DMSO; Sigma, 276855).

### Preparation of CORM-2 and Inactive CORM-2

Tricarbonyldichlororuthenium (II) dimer (CO-releasing molecule-2, CORM-2) purchased from Sigma-Aldrich Canada (Cat #: 288144, Oakville, Ontario, Canada) was dissolved in DMSO to make a 50 mM stock solution and kept at −20°C for short-term storage or −80°C for long-term storage respectively. Inactive CORM-2 (iCORM-2) was prepared by dissolving CORM-2 in DMSO to make 1 mM solution, and kept for 3 days at room temperature. It was diluted with equal amount of DMEM and kept at 37°C for 24 h before use.

### Culture of Primary Cells

All animal experiments were approved by the Institutional Animal Care and Use Committees of the University of Manitoba (Protocol #: 09-053). Cultures of oligodendrocytes, microglia, and astrocytes were prepared from postnatal day 1 rat pups. Microglia and oligodendrocyte precursor cells (OPCs) were prepared using protocols described previously (6–8). Briefly, rat pups were sacrificed by decapitation. Cerebral cortices were dissected out and mechanically dissociated. The cells were then grown in DMEM, supplemented with 20% fetal bovine serum. After 10 days, the flasks containing the cells were shaken at 220 rpm for 1 h to collect microglia which were further cultured in DMEM supplemented with 10% FBS. The flasks containing the remaining cells were then shaken at 220 rpm overnight to dislodge loosely attached OPCs. OPCs were further purified from astrocytes and microglia by being placed in uncoated tissue culture dishes for 1 h. The non-adherent cells were collected and cultured in a chemically defined medium (CDM) containing 4 mM L-glutamine, 1 mM sodium pyruvate, 0.1% bovine serum albumin, 50 μg/ml Apo-transferrin, 5 μg/ml insulin, 30 nM sodium selenite, 10 nmol/L D-biotin, and 10 nM hydrocortisone. This CDM was supplemented with 10 ng/ml PDGF-AA and 10 ng/ml bFGF (PeproTech, Rocky Hill, NJ, USA). Cultures of astrocytes were performed using our standard protocols as described previously (9).

Primary cortical neuronal cultures were prepared from E18 rat fetuses as described previously (10). Briefly, neurons were plated in neurobasal medium (Cat# 21103049, Thermo Fisher Scientific, Pittsburgh, PA, USA) supplemented with 5 mmol/L HEPES, 1.2 mmol/L glutamine, 10% fetal bovine serum, 2% B27 (Cat# 17504044, Thermo Fisher Scientific, Pittsburgh, PA, USA), and 25 μg/ml gentamicin at a density of 1x104 cells/cm^2^ on chamber slides coated with poly-D-lysine. The medium was replaced with neurobasal medium without fetal bovine serum after 24 h. After 7 days in culture, glutamine was removed from the medium.

Well-differentiated neurons, astrocytes, microglia, and oligodendrocytes at a density of 5 × 10^4^ cells/cm^2^ were then used for treatments with CORM-2 or iCORM-2. Cells treated with the same amount of dissolvent (DMSO) were used as controls.

Oligodendrocyte-neuron co-culture was performed from rat E16 spinal cord-derived cells as described previously (11), with modifications. Briefly, spinal cords from embryos were collected in a petri dish containing HBSS (without Ca^2+^ and Mg^2+^). After carefully removing the meninges, the spinal cord tissue was cut into small pieces with a surgical blade. The minced tissue was then transferred into a 15-ml centrifuge tube with 1 ml StemPro Accutase and incubated for 20 min at 37°C for enzymatic dissociation. The dissociated cell suspension was then passed through a 40-μm cell strainer. Cells were then seeded on poly-L-lysine-coated coverslips at a density of 0.4 × 10^5^/cm^2^. After 2 h adhesion, the plating medium (50% DMEM, 25% HBSS, and 25% horse serum) was replaced with differentiation medium (DMEM and modified ITS culture supplement). At DIV (Days *in vitro*) 14, insulin was excluded from the culture system. The cultures were exposed to CORM-2 at 100 µM from DIV 21 for 7 days.

### MTT and WST-1 Assays

The assays were performed in accordance with the manufacturer’s instructions. Briefly, a total of approximately 0.25 × 10^5^ cells were cultured in each well of a 96 well plate in a final volume of 100 µl. Triplicates of each condition were prepared. For MTT assay, After CO exposure, MTT (Thiazolyl blue tetrazolium bromide, MTT; Cat #: M5655, Sigma-Aldrich Canada, Oakville, Ontario) or WST-1 (#ab 155902, Abcam, Cambridge, UK) was added to each well, and the plates were incubated in a 5% CO_2_ atmosphere at 37°C for 4 h. Then, the solution was removed, followed by the addition of 200 μl of DMSO to each well. The absorbance was detected at 570 nm for MTT assay or 460 nm for WST-1 assay on a plate reader. The experiments were performed in triplicate in three individual experiments.

### Cell Death Analysis

PC12 cells at a density of 2 × 10^4^ cells/well in 96-well plates were exposed to CORM-2 at different doses (50, 100, 150, 200 μM). A 1:1,000 dilution of Hoechst 33258 stock in DPBS (diluted fresh each time; final concentration was 0.12 μg/ml) was added to the wells. The cells were incubated in the dark for 15 min at room temperature. Then, they were rinsed five times with DPBS, and treated with a 1:1,000 dilution of propidium iodide (final concentration was 0.3 μg/ml), followed by incubation in the dark for 3 min at room temperature. The experiments were performed in triplicate in three individual experiments. Images of ten random fields were taken on a fluorescence microscope (TE2000-E, Nikon).

### Immunohistochemistry

Immunofluorescence staining was performed using our standard protocols (12, 13). Co-culture samples were washed three times with PBS, blocked with 1% BSA PBST for 30 min at room temperature, and then washed with PBS containing 0.25% Triton X-100. The samples were incubated with the following primary antibodies: polyclonal pNF-H (1:500), monoclonal PLP (Sigma G3893, St Louis, MO, USA, 1:500). After being washed three times, the samples were incubated with Alexa Fluor^®^ conjugated secondary antibodies for 2 h at room temperature. The samples were then incubated with Hoechst 33342 for identification of the nuclei (Sigma B2261, St Louis, MO, USA, at a final concentration of 1 μg/ml). Negative controls were performed by using PBS instead of the primary antibodies. Axon myelination was calculated as the ratio of PLP-covered axons to total axons.

### Statistical Analysis

Statistical analysis was performed with one-way ANOVA to determine the statistical significance of experimental factors, followed by Tukey’s *post hoc* test. Comparisons between two groups were analyzed using Student’s t-test. P < 0.05 was considered significantly different. Data are presented as means ± SEM.

## Results

**Figure 1:**
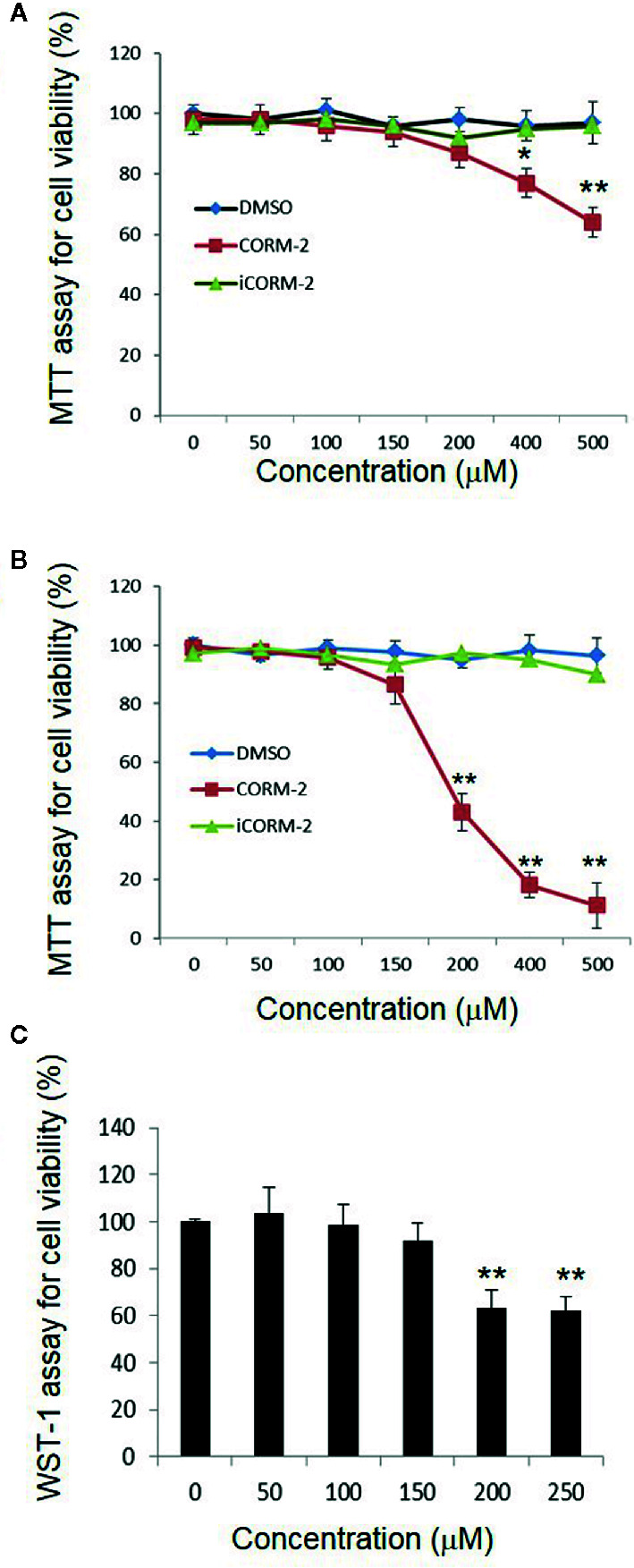
Direct cytotoxicity of carbon monoxide to PC12 cells. PC12 cells were exposed to iCORM-2 and CORM-2 at indicated concentrations for 6 h **(A)** or 24 h **(B)**, and cell viability was determined by MTT assay. Cells exposed to DMSO were included as controls. Readings of MTT assays were normalized to 100% for cells treated with DMSO. For WST-1 assay **(C)**, PC12 cells were exposed for 24 h. Shown are mean +/- SD from triplicate samples from three independent experiments. *P < 0.05, **P < 0.01.

**Figure 2:**
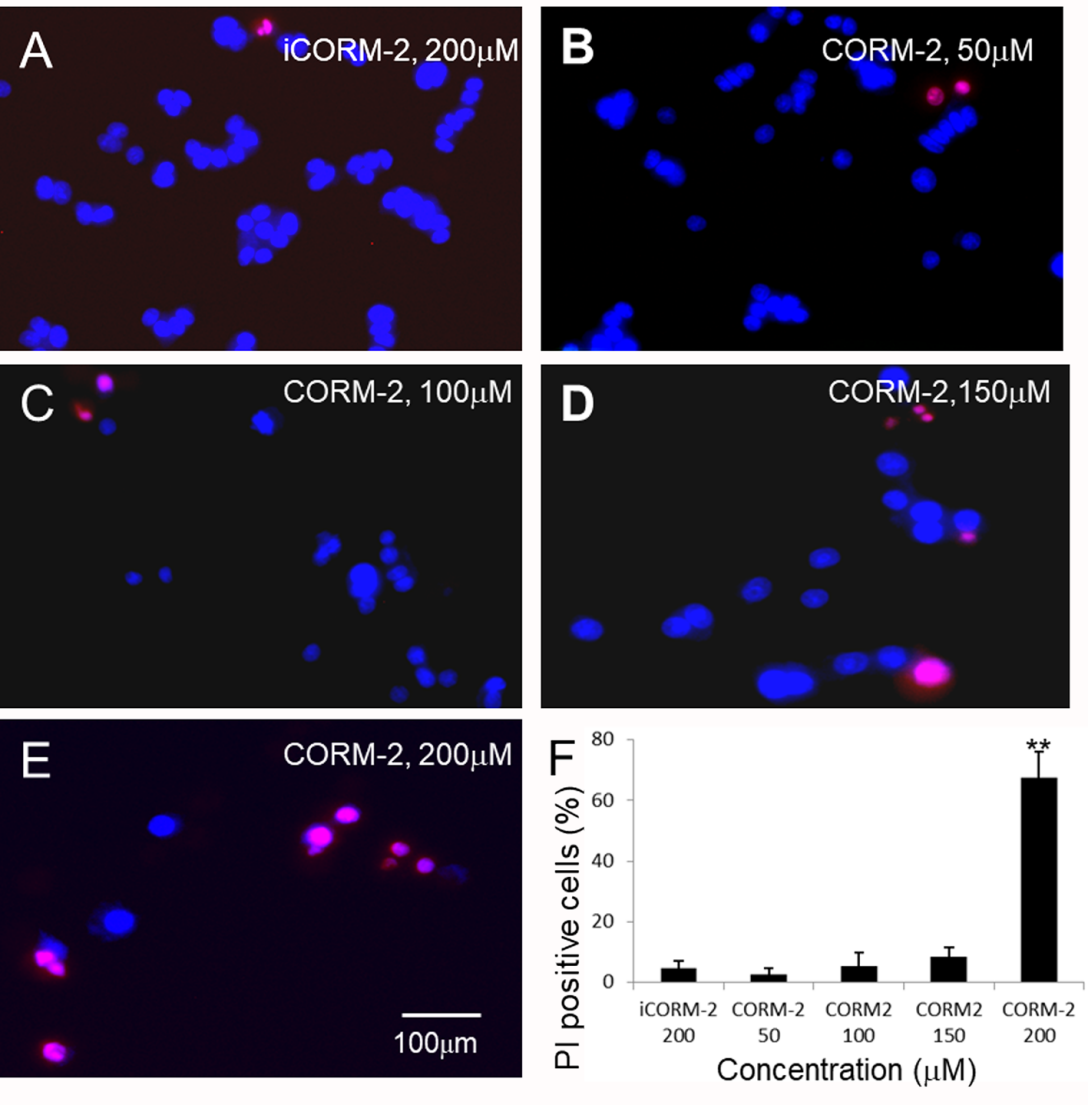
Propidium iodide (PI) staining analysis of PC12 cells. **(B–E)** PC12 cells were exposed to CORM-2 at indicated concentrations for 24 h. Cells exposed to iCORM-2 **(A)** were used as controls. **(F)** Number of PI-positive cells. Shown are mean +/- SD from 10 randomly selected fields of each group. Scale bar = 100 μm.

We first determined direct toxicity of CO in an *in vitro* cell culture model using CORM-2 as the CO donor. Cultured PC12 cells were exposed to CORM-2 at concentrations from 50 μM to 500 µM for 6 or 24 h. MTT and WST-1 assays were used to evaluate cell viability. The MTT assay is based on the conversion of water soluble MTT 3-(4,5-dimethylthiazol-2-yl)-2,5-diphenyltetrazolium bromide) compound to an insoluble formazan product in viable cells. The WST-1 assay is based on the cleavage of the tetrazolium salt WST-1 to formazan by cellular mitochondrial dehydrogenases. As shown in **Figure 1A**, the PC12 cells exposed to CORM-2 for 6 h at concentrations lower than 200 µM maintained similar viability as compared to the controls. At concentrations higher than 400 µM, CORM-2 caused a significant reduction of the cell viability. The minimum toxic concentration of CORM-2 reduced to 200 µM when the PC12 cells were exposed for 24 h (**Figures 1B, C**). Propidium iodide (PI) is a membrane-impermeant nucleic acid intercalator and stains membrane-permeable dying cells. Consistent with significant reduction of viability in the PC12 cells, PI staining revealed that CORM-2 at concentrations higher than 200 µM induced significant cell death (**Figure 2**).

**Figure 3:**
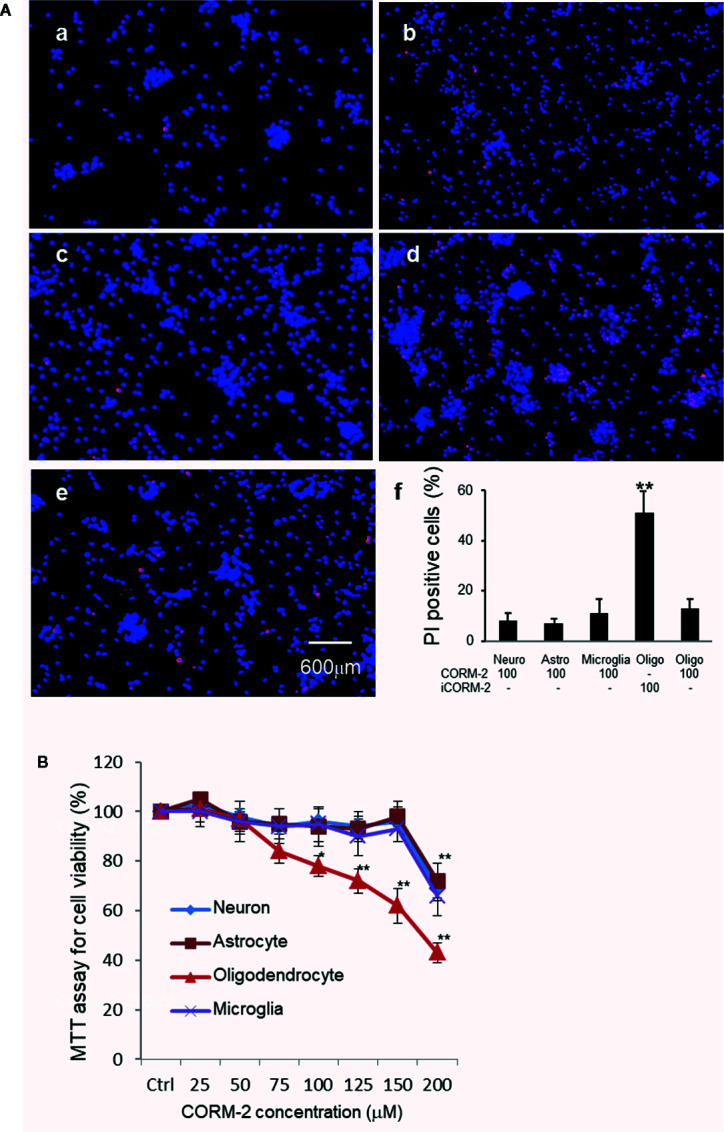
Selective susceptibility of oligodendrocytes to CO toxicity. **(A)** PI staining of primary neurons (a), astrocytes (b), microglial cells (c), and oligodendrocytes (d) after exposure to CORM-2 at 100 µM for 24 h. Oligodendrocytes treated with iCORM-2 at 100 µM for 24 h (e) were used as control. (f), Percentage of PI-positive cells. Shown are mean +/- SD from 10 randomly selected fields of each group under the same mangafication. Scale bar in e = 600 μm. **(B)** MTT analysis of the CORM-2-induced cytotoxicity in neurons, microglia, astrocytes, and oligodendrocytes. Cells were exposed to CORM-2 at indicated concentrations or iCORM-2 (200 µM) as the control for 24 h. *P < 0.05; **P < 0.01.

We then determined cytotoxicity of carbon monoxide to major cells in the brain, namely neurons, oligodendrocytes, astrocytes, and microglia. Well-differentiated cortical neurons, microglia, astrocytes, and oligodendrocytes were treated with CORM-2 at concentrations from 25 to 200 µM for 24 h. Cells treated with iCORM-2 at 200 µM were included as controls. MTT assays showed that the minimum toxicity concentration of CORM-2 to neurons, astrocytes and microglia was at 200 µM, the same as that to the PC-12 cells. Interestingly, oligodendrocytes started to show a reduced viability when exposed to CORM-2 at the concentration of 75 µM (**Figure 3**). The reduction in viability became statistically significant at 100 µM and very significant at 125 µM or higher.

**Figure 4:**
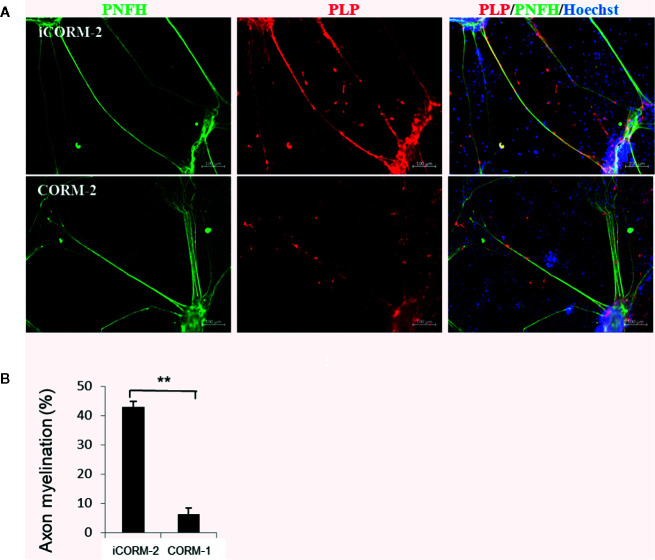
Effect of CO on myelination. **(A)** CO inhibited myelin production but had no effects on growth of axons. The *in vitro* model of myelination established from co-culture of spinal cord neurons and oligodendrocytes was exposed to CORM-2 or iCORM-2 at 100 mM for 7 days. Myelination was detected with immunostaining with antibodies to phosphorylated neurofilament H subunit (pNFH) and myelin proteolipid protein (PLP). **(B)** Myelination determined by coverage of myelin on axons. Shown are mean +/- SD from 10 randomly selected fields. **P < 0.01.

We then determined the effects of CO released from CORM-2 on myelination. Co-culture of neurons and oligodendrocytes from spinal cord facilitates formation of myelin on axons by 3 weeks. On DIV 21, the co-cultures were exposed to CORM-2 for 7 days at 100 µM, a concentration that was known to be tolerated in neurons but toxic to oligodendrocytes. The CORM-2-containing medium was changed daily. iCORM-2 at 100 µM was used as the controls. As shown in **Figure 4**, CORM-2 at 100 µM remarkably inhibited the production of the myelin proteolipid protein and significantly reduced the coverage of myelin on axons.

## Discussion

The present study provides evidence for direct cytotoxicity of carbon monoxide released from CORM-2. From our data, we conclude that oligodendrocytes, compared to other brain cells, are particularly susceptible to CO poisoning. The results help suggest that DNS may result from direct cytotoxicity of CO.

Previous studies on CO poisoning are largely referenced to hypoxic damage in the brain, since CO blocks the binding of oxygen to hemoglobin which could induce ineffective delivery of oxygen. Since carbonylation is known to occur in all the cells, excessive CO would likely exert a direct cytotoxicity. It is reported that carbonyl damage to lipids, sugars, and amino acids can irreversibly alkylate and crosslink proteins (14).

CORM-2 is a CO donor. Previous studies have shown that CORM-2 is stable in DMSO, however, CO is released in physiological solutions and biological fluids such as PBS, Krebs buffer, cell culture media, and human blood plasma, all at pH 7.4. In these conditions, one mole equivalent of CO is released quite rapidly. Additionally, there are indications that the remaining CO may be released at a later time (15), so we prepared iCORM-2 by dissolving CORM-2 in DMSO for 3 days at room temperature then double diluted it with DMEM and left it for more than 24 h at 37°C under a 5% CO_2_ humidified atmosphere. In a pilot experiment (data not present here), we detected CO released from CORM-2 in medium at 24 h at both low and high concentrations of CORM-2, with high concentrations of CORM-2 leading to higher levels of CO release.

Low concentrations of CORM-2 have been used to analyze CO’s protective effects against cell apoptosis and inflammation (16, 17). In our study, we confirm that CO at low concentrations did slightly increase viability of PC12 and neural cells. At concentrations higher than 200 µM, CORM-2 inhibited viability and induced significant cell death of PC12 cells, neurons, astrocytes and microglia. Interestingly, the minimum toxicity concentration for oligodendrocytes was at 75 µM, a concentration that was well tolerated in all the cell types tested, indicating that oligodendrocytes are particularly vulnerable to PO poisoning. This is in agreement with previous observations that oligodendrocytes are the most vulnerable cells in the brain in response to hypoxic and oxidative stresses (8, 18).

Myelin in the CNS is formed by oligodendrocytes developed from oligodendrocyte precursor cells (OPCs). In response to neuronal activity, OPCs proliferate, migrate to contact axons, then differentiate into mature oligodendrocytes to generate myelin (19, 20). Demyelination, characterized by loss of the myelin sheath and oligodendrocyte cell death (21), is common in many neurological and psychiatric disorders. DNS following CO poisoning features white matter damages as suggested by neuroimaging studies in patients with DNS (5). It is tempting to speculate that acute damages of CO poisoning are largely caused by severe hypoxia while the delayed damages contributing to white matter damages in DNS after CO exposure may result from direct cytotoxicity of CO. CO-induced toxicity may include acidosis, nitrative stress, oxidative stress, inflammation, and apoptosis of oligodendrocytes (22). Further studies are needed to reveal mechanisms of CO cytotoxicity and high susceptibility of oligodendrocytes to CO poisoning.

## Data Availability Statement

All datasets generated for this study are included in the article/**Supplementary Material**.

## Ethics Statement

The animal study was reviewed and approved by The Institutional Animal Care and Use Committee of the University of Manitoba.

## Author Contributions

XT conducted the experiments and wrote draft of the manuscript. TG, YG, and GZ conducted the experiments. JK designed the experiments and revised the manuscript.

## Funding

The study was supported by grants from the Health and Family Planning Commission of Hebei Province (20170179), the Department of Education of Hebei Province (Z2017027) and ALS Canada/Brain Canada.

## Conflict of Interest

The authors declare that the research was conducted in the absence of any commercial or financial relationships that could be construed as a potential conflict of interest.
